# The transdiagnostic role of event-related rumination on internalizing and externalizing symptoms during the pandemic: a two-wave longitudinal study

**DOI:** 10.3389/fpsyg.2025.1421958

**Published:** 2025-03-18

**Authors:** Bin-Na Kim, Hyo Shin Kang, Jungkyu Park

**Affiliations:** ^1^Department of Psychology, Gachon University, Seongnam, Republic of Korea; ^2^Department of Psychology, Kyungpook National University, Daegu, Republic of Korea

**Keywords:** rumination, transdiagnostic, internalizing, externalizing, psychopathology

## Abstract

**Background:**

Rumination is a well-established transdiagnostic vulnerability. However, few studies have explored the transdiagnostic role of event-related rumination. Moreover, there is a paucity of longitudinal studies clarifying the temporal precedence of event-related rumination. Therefore, this study aimed to longitudinally examine the mediating paths of event-related rumination between perceived stress and diverse symptomatic dimensions.

**Methods:**

A representative sample of Korean adults (*N* = 316) was recruited online and they completed a package of self-reported measures twice over a one-year period. Using prospective two-wave data collected during the pandemic, longitudinal indirect effects were examined using the hypothesized path model.

**Results:**

As expected, intrusive rumination acted as a transdiagnostic mediator in both internalizing and externalizing psychopathology and was positively associated with all subsequent symptom dimensions, except mania. Meanwhile, the beneficial role of deliberate rumination was less-transdiagnostic.

**Conclusion:**

These initial findings suggest that event-related rumination could be considered a transdiagnostic mediator and a target for prevention and intervention to maintain mental health during and after the pandemic.

## 1 Introduction

Rumination is a representative form of repetitive negative thinking (RNT), a well-known transdiagnostic vulnerability for a wide array of both internalizing and externalizing psychopathologies including depression, anxiety, substance abuse, binge eating, insomnia, psychosis, and self-injurious and impulsive behaviors (Johnson et al., 2016; McEvoy et al., 2013; McLaughlin et al., 2014; Nolen-Hoeksema and Watkins, 2011). Ample evidence suggests that rumination worsens the severity and duration of psychiatric symptoms by intensifying physiological stress responses (e.g., cardiovascular activity and cortisol levels) and impairing attentional control, executive memory, and instrumental behavior (Hsu et al., 2015; Watkins and Roberts, 2020). Rumination was primarily studied in relation to depression in earlier works, thereby once labeled as depressive rumination (Nolen-Hoeksema, 1991). Later, other forms of ruminative thinking, focusing on different contents albeit a similar process, were added to the literature, such as angry rumination (Sukhodolsky et al., 2001) or event-related rumination (Cann et al., 2011).

In particular, event-relation rumination was proposed as a cognitive process involved in the aftermath of traumatic or highly stressful life events, which conveys differential implications for subsequent adaptation (Cann et al., 2011). Analogous to maladaptive brooding and the putatively adaptive reflection distinction in depressive rumination (Treynor et al., 2003), intrusive rumination (IR) refers to the involuntary and uncontrollable invasion of repetitive thoughts about an event, whereas deliberate rumination (DR) is characterized by a more constructive and problem-focused thought that makes meaning of the event. Cann et al. (2011) postulated that although they are both normal by-products that can emerge in an individual’s struggle during major life crises, IR would lead to continued distress and failed coping, whereas DR would be conducive to later adaptation by fostering posttraumatic growth (PTG) over time. Recent meta-analyses have demonstrated that this differential relationship of event-related rumination subtypes is well replicated with moderate effect sizes (Allen et al., 2022; Szabo et al., 2017). In most studies, adaptational outcomes were limited to post-traumatic stress disorder (PTSD) symptoms and/or PTG. Generally speaking, IR was positively related to PTSD symptoms, whereas DR was positively associated with PTG. And this was also consistent in a few empirical studies (Ikizer et al., 2021; Wall et al., 2023) investigating the psychological adaptation pattern towards the coronavirus disease 2019 (COVID-19) crisis, which has been a salient stressful event, causing a drastic increase in diverse mental health problems globally (Xiong et al., 2020).

However, there is a lack of research investigating the role of event-related rumination from a transdiagnostic perspective. To the best of our knowledge, Squires et al. (2022) were among the first to delineate the transdiagnostic mechanism of event-related rumination beyond PTSD or PTG. They showed that IR was a partial mediator of the association between non-infection pandemic stress and depression and anxiety severity. By contrast, DR did not demonstrate significant indirect effects between pandemic stress and both symptom severities. However, cross-sectional design limited their explanation of directionality between event-relation rumination and symptom. A dearth of longitudinal studies that can confirm the temporal precedence of event-related rumination has been highlighted as a common limitation of the existing literature in this area (Allen et al., 2022). Additionally, as they included only two symptom dimensions, both belonging to internalizing psychopathology, it was necessary to widen the scope of symptomatic outcomes to provide a more comprehensive transdiagnostic account. Currently, it is unknown whether event-related rumination is predictive of externalizing psychopathology.

Therefore, this study attempted to longitudinally explore the mediating pathways of event-related ruminations between perceived stress and both internalizing and externalizing psychopathology, utilizing two-wave data collected during the recent pandemic. Based on the more consistent association between IR and psychiatric symptoms, we expected IR to be a transdiagnostic mediator between perceived stress and both internalizing and externalizing psychopathologies. Given that the role of DR in preventing or mitigating negative outcomes has been relatively weak in prior research (Kang and Kim, 2021; Squires et al., 2022), we anticipated that the mediating role of DR would be less transdiagnostic than that of IR.

## 2 Methods

### 2.1 Participants and procedures

Baseline (Time 1, T1) data were collected in August 2020 from the Greater Daegu area, where the first massive outbreak occurred in South Korea. A total of 316 adults (*M* = 43.27 years, *SD* = 12.61, 50.6% female) were recruited using stratified sampling in terms of age and gender for representativeness. Follow-up (Time 2, T2) data collection was conducted in August 2021, when the delta variant of COVID-19 caused an overwhelming increase in infection and hospitalization nationwide, and 44.6% of the original participants were retained (*N* = 141, *M* = 49.1 years, *SD* = 10.6, 42.6% female). There was no significant difference between the retained and the dropped-out participants in terms of COVID-19-related experiences at baseline including a COVID-19 diagnosis. After obtaining informed consent, the participants answered a package of online self-report questionnaires as part of a longitudinal research project to investigate individual differences in psychological adaptation to the pandemic. All procedures and materials were approved by the Institutional Review Board of Kyungpook National University (KNU-2020-0054/KNU-2021-0119).

### 2.2 Measures

#### 2.2.1 Demographics and COVID-19-related variables

Data regarding age, gender, educational level, and marital status were collected. Questions regarding COVID-19-related experiences were also included. In addition to asking whether participants had experienced a COVID-19 infection (diagnosis of themselves, family members, or close friends), screening tests, self-quarantine, or vaccination, two five-point Likert scales (from 1 = not at all to 5 = very much) were used to measure the subjective severity of COVID-19-related experiences [disruption in daily life, *M* = 3.84, *SD* = 0.78 and perceived traumatic experience (*M* = 2.94, SD = 0.10)], adapted from García et al. (2015).

#### 2.2.2 Korean version of the perceived stress scale (K-PSS)

The PSS is a self-report questionnaire that assesses the degree of an individual’s perceived level of stress in daily life (Cohen et al., 1983), and was validated in Korean by Lee et al. (2012). It comprises 10 items rated on a five-point Likert scale (0 = never to 5 = very often). The internal consistency was appropriate in this study (Cronbach’s *α* = 0.76).

#### 2.2.3 Korean version of the event-related rumination inventory (K-ERRI)

The ERRI was developed to assess two types of rumination during major life crises: intrusive and deliberate rumination (Cann et al., 2011). The K-ERRI was used in this study (Ahn et al., 2013), consisting of 20 items (10 items for intrusive and deliberate rumination) rated on a 4-point Likert scale (0 = not at all to 3 = often). To focus on event-related rumination in response to the pandemic, the researchers slightly changed the wording of the phrase “during the weeks immediately after the event” into “during COVID-19.” Internal consistency was excellent in this study (Cronbach’s *α*: intrusive rumination = 0.96; deliberate rumination = 0.91).

#### 2.2.4 Korean version of the center for epidemiologic studies-depression scale (K-CESD)

The CES-D is a well-known 20-item self-administered questionnaire developed by Radloff (1977) to assess depression severity in the general population. Each item is answered on a 4-point Likert scale (0 = rarely to 3 = most or all of the time). The Korean version was validated by Chon et al. (2001) and Cronbach’s *α* in this study was 0.88.

#### 2.2.5 Mental health screening tool for suicide risk (MHS)

Yoon et al. (2020) developed the MHS as an ultra-brief measure to assess suicide risk. It includes four items (will to live, suicidal thoughts, suicidal plans, and history of suicide attempts) on a 5-point Likert scale. The internal consistency in this study was excellent (Cronbach’s *α* = 0.90).

#### 2.2.6 Korean brief symptom inventory (K-BSI)

The BSI is a widely used self-report questionnaire that assesses psychological distress, including depression, anxiety, and somatization (Derogatis, 2001). It contains 18 items rated on a 5-point Likert scale (1 = not at all to 5 = very much). The K-BSI was validated by Park et al. (2012). We used scores from the two subscales, anxiety and somatization (Cronbach’s *α* = 0.90, 0.91 respectively).

#### 2.2.7 Korean version of the state–trait anger expression inventory (STAXI-K)

It was developed to measure individual experiences of anger, including state anger, trait anger, anger suppression, anger expression, and anger control (Spielberger et al., 1988). The Korean version was validated by Chon et al. (1998), and 10 items related to state anger were used in this study. Each item was answered on a 4-point Likert scale (from 1 = not at all to 4 = very much), and the internal consistency was excellent (Cronbach’s *α* = 0.95).

#### 2.2.8 Korean version of the Altman self-rating mania scale (K-ASRM)

The ASRM is a brief self-report tool measuring the severity of manic symptoms, developed by Altman et al. (1997). Kim and Kwon (2017) validated the Korean version of this questionnaire. It comprises five items (elevated mood, increased self-esteem, decreased sleep need, pressured speech, and increased activity) rated on a 5-point Likert scale (from 0 = symptoms not present to 4 = symptoms present to a severe degree). The internal consistency was moderate in this study (Cronbach’s *α* = 0.77).

### 2.3 Statistical analysis

**Figure 1:**
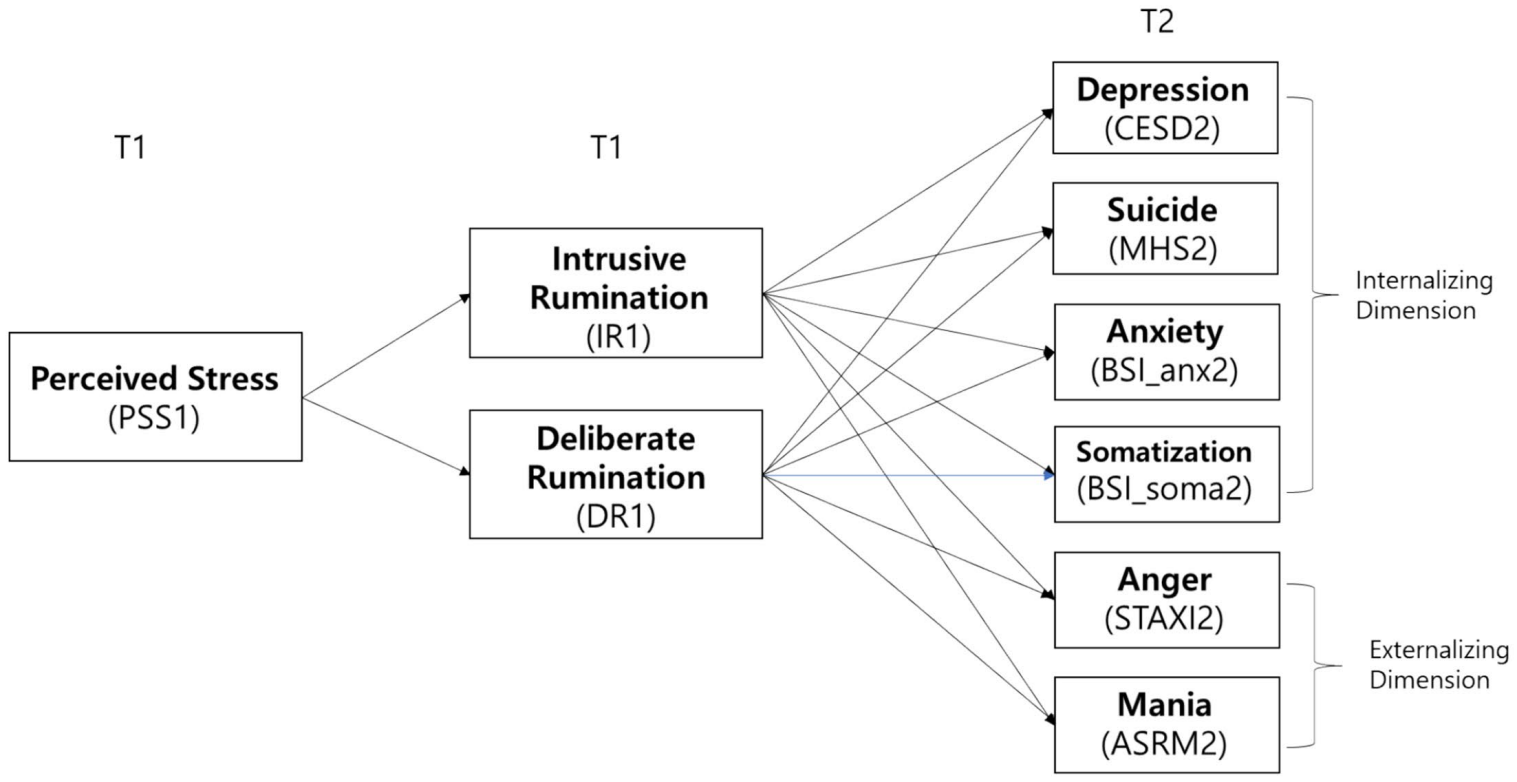
The hypothesized model, age, gender, and the subjective severity of COVID-19-related experience were controlled for as covariates.

We hypothesized that the perceived stress at T1 is associated with IR and DR at T1, and these variables are related to outcome variables at T2, including depression, suicidal tendencies, anxiety, somatization, anger, and mania, as depicted in Figure 1. While estimating the path coefficients representing the associations between the variables, we controlled for gender, age, and subjective severity of COVID-19-related experiences. Moreover, the path coefficients from mediators at T1 to outcome variables at T2 were estimated while controlling for baseline symptoms. This was achieved by including the outcome variables at T1—such as depression, suicidal tendencies, anxiety, somatization, anger, and mania—as control variables in the model.

Based on the path coefficient estimates, we computed 12 indirect effects. These effects revealed that perceived stress indirectly influenced the outcome variables through one of the rumination processes. To evaluate the significance of these indirect effects, 95% CIs for the average indirect effects were constructed using Monte Carlo simulation procedure. This approach accurately reflects the asymmetric nature of the indirect effect’s sampling distribution by producing empirical sampling distributions of the path coefficients used to calculate the indirect effect (Preacher and Selig, 2012).

## 3 Results

After descriptive and correlational analyses (Table 1), standard path coefficient estimates in the hypothesized model were calculated. As shown in Table 2, perceived stress at T1 was positively related to both IR (*β* = 0.68, *p* < 0.01) and DR (*β* = 0.38, *p* < 0.01) at the same time point. Also, IR exhibited positive associations with several outcome variables at T2, such as depression (*β* = 0.61, *p* < 0.01), suicide (*β* = 0.56, *p* < 0.01), anxiety (*β* = 0.49, *p* < 0.01), somatization (*β* = 0.35, *p* < 0.001), anger (*β* = 0.45, *p* < 0.001), except for mania (*β* = −0.25, *p* < 0.01). However, DR was negatively associated with depression (*β* = −0.21, *p* < 0.05), suicide (*β* = −0.31, *p* < 0.01) and anxiety (*β* = −0.26, *p* < 0.01) at T2.

**Table 1:** Descriptive and correlational analyses (*N* = 141).

Variable	Mean	SD	1	2	3	4	5	6	7	8	9	10	11
1. Gender	-	-											
2. Age	49.14	10.57	−0.16										
3. COVID-19	3.67	0.76	0.19^*^	−0.24^**^									
4. PSS1	19.04	4.32	0.01	−0.15	0.34^**^								
5. IR1	11.50	7.45	0.06	−0.14	0.28^**^	0.70^**^							
6. DR1	13.04	5.93	−0.06	−0.18^*^	0.29^**^	0.46^**^	0.66^**^						
7. CESD2	18.56	10.62	−0.01	0.06	0.10	0.55^**^	0.51^**^	0.21^**^					
8. MHS2	1.62	2.89	0.02	0.02	0.11	0.38^**^	0.41^**^	0.08	0.56^**^				
9. BSI_anx2	13.81	5.70	−0.02	0.14	0.11	0.47^**^	0.42^**^	0.20^**^	0.79^**^	0.53^**^			
10. BSI_soma2	11.43	4.76	−0.09	0.21^*^	−0.02	0.34^**^	0.25^**^	0.06	0.72^**^	0.47^**^	0.85^**^		
11. STAXI2	15.12	6.34	−0.08	0.17	0.09	0.33^**^	0.31^**^	0.05	0.73^**^	0.39^**^	0.67^**^	0.64^**^	
12. ASRM2	2.81	2.86	−0.22^**^	−0.29^**^	−0.07	−0.30^**^	−0.19^*^	−0.04	−0.31^**^	−0.11	−0.36^**^	−0.24^**^	−0.19^*^

COVID-19, subjective severity of COVID-19; PSS1, perceived stress at T1; IR1, intrusive rumination at T1; DR1, deliberate rumination at T1; CESD2, depression at T2; MHS2, suicide at T2; BSI_anx2, anxiety at T2; BSI_soma2, somatization at T2; STAXI2, anger at T2; ASRM2, mania at T2; ^*^p < 0.05, ^**^p < 0.01.

**Table 2:** Standard path coefficient estimates in the hypothesized model.

	Intrusive Rumination (T1) (Mediator 1)		Deliberate Rumination (T1) (Mediator 2)	
	*β*	S.E.	*β*	S.E.
Independent variable				
Perceived stress (T1)	0.68^**^	0.05	0.38^**^	0.07
Outcome variable				
Depression (T2)	0.61^**^	0.07	−0.21^*^	0.09
Suicide (T2)	0.56^**^	0.08	−0.31^**^	0.09
Anxiety (T2)	0.49^**^	0.09	−0.12	0.10
Somatization (T2)	0.35^**^	0.09	−0.15	0.10
Anger (T2)	0.45^**^	0.09	−0.26^*^	0.10
Mania (T2)	−0.25^**^	0.10	0.07	0.10

^*^p < 0.05, ^**^p < 0.01.

Subsequently, the longitudinal indirect effects were calculated using the path coefficient estimates. As displayed in Table 3, perceived stress and most of the outcome variables were positively related through IR, including depression (estimates = 1.14, [0.762, 1.567]), suicide (estimates = 0.29, [0.186, 0.408]), anxiety (estimates = 0.47, [0.274, 0.695]), somatization (estimates = 0.29, [0.118, 0.480]) and anger (estimates = 0.50, [0.278, 0.760]), whereas only mania was negatively associated through IR (estimates = −0.12, [−0.222, −0.021]). Conversely, negative associations were found between perceived stress and three outcome variables through DR: depression (estimates = −0.21, [−0.455, −0.019]), suicide (estimates = −0.09, [−0.164, −0.030]), and anxiety (estimates = −0.16, [−0.322, −0.032]).

**Table 3:** Indirect effect estimates in the hypothesized model.

Indirect effects	Estimates	S.E.	*p*	95% Confidence interval
1. PSS1 → IR1 → CESD2	1.14	0.21	0.00	[0.762, 1.567]
2. PSS1 → IR1 → MHS2	0.29	0.06	0.00	[0.186, 0.408]
3. PSS1 → IR1 → BSI_anx2	0.47	0.11	0.00	[0.274, 0.695]
4. PSS1 → IR1 → BSI_soma2	0.29	0.09	0.00	[0.118, 0.48]
5. PSS1 → IR1 → STAXI2	0.50	0.12	0.00	[0.278, 0.760]
6. PSS1 → IR1 → ASRM2	−0.12	0.05	0.01	[−0.222, −0.021]
7. PSS1 → DR1 → CESD2	−0.21	0.11	0.02	[−0.455, −0.019]
8. PSS1 → DR1 → MHS2	−0.09	0.03	0.00	[−0.164, −0.030]
9. PSS1 → DR1 → STAXI2	−0.16	0.07	0.01	[−0.322, −0.032]

PSS1, perceived stress at T1; IR1, intrusive rumination at T1; DR1, deliberate rumination at T1; CESD2, depression at T2; MHS2, suicide at T2; BSI_anx2, anxiety at T2; BSI_soma2, somatization at T2; STAXI2, anger at T2; ASRM2, mania at T2; ^*^p < 0.05, ^**^p < 0.01.

## 4 Discussion

This study aimed to examine whether perceived stress indirectly influences a wide range of psychopathologies through two types of event-related rumination, adopting a transdiagnostic perspective. Consistent with previous studies demonstrating the maladaptive function of IR (Squires et al., 2022; Zhou and Wu, 2016), the indirect effects of IR (T1) were significant in all symptomatic dimensions (T2) belonging to both internalizing and externalizing psychopathologies. This indicates that IR can be considered as a transdiagnostic mediator, as expected. The direction of this relationship differed depending on the symptoms. IR was positively related to almost all symptoms 1 year later, *except* manic symptoms. That is, engaging more in IR in the acute phase of the pandemic subsequently led to higher levels of depression, suicide risk, anxiety, somatic symptoms, and anger but a lower level of mania symptoms.

Given that the core characteristics of RNT, including IR, are passive and unproductive (Watkins, 2008), it is understandable that IR worsened the intensity of various internalizing psychopathologies, as was the case in the brooding subtype of depressive rumination (García et al., 2017). With respect to externalizing psychopathology, IR aggravated anger but reduced manic symptoms over time. This may be owing to the different underlying mechanisms. The development of mania has been considered more motivational in nature (Johnson et al., 2012) than the direct by-products of cognitive evaluation, as compared to anger, which is closely associated with cognitive appraisals (Sukhodolsky et al., 2001), although this does not mean that manic symptoms are independent of cognitive appraisal. In fact, the relationship between RNT and manic symptoms is an under-examined topic, and only recently has one cross-sectional study attempted to address this gap (Samtani et al., 2022). Though they did not include the ERRI, the correlation between brooding, which is analogous to IR in the ERRI, and manic symptom was also negative (*r* = −0.11, *p* < 0.01), consistent with ours. Further research is necessary to clarify why RNT, including IR, are negatively related to manic symptoms.

Next, the current results demonstrate that the mediating role of DR was protective and less transdiagnostic, which is consistent with our expectations. Originally, DR was conceptualized as a putatively adaptive type of event-related rumination, which co-occurs with maladaptive IR in the earlier phase, but may eventually lead to PTG over time (Cann et al., 2011). However, there have been mixed findings on whether this prediction is empirically supported (Allen et al., 2022; Kang and Kim, 2021; Taku et al., 2021). This study provided longitudinal evidence that DR acts as a protective factor, which may have been obscured in previous cross-sectional studies. But the beneficial effects of DR were less transdiagnostic, affecting a narrower range of symptom dimensions longitudinally. DR (T1) prospectively mediated the links between perceived stress (T1) and the three symptom dimensions (T2) of depression, suicide risk, and anger symptoms. Conversely, DR did not exhibit significant indirect effects on anxiety, somatization, or mania symptoms.

Thus, these results suggest that beneficial effects of DR are valid in symptomatic domains primarily involving negative evaluations towards self or others (du Pont et al., 2018). In fact, DR is conceptually adjacent to *positive reevaluation* of what happened, as it is a constructive meaning-making process (Taku et al., 2021). Therefore, it is probable that DR counteracts the adverse effects of negative evaluations underlying depression- or anger-related psychopathology. Meanwhile, different thematic or temporal foci in anxiety- or somatization-related RNT seem less likely to be effectively tackled by DR (Marcus et al., 2007; Watkins et al., 2005).

This study has several theoretical and clinical implications. From a theoretical perspective, we extended the existing literature on rumination by exploring the transdiagnostic role of event-related rumination. As noted previously, few studies have addressed this issue (Squires et al., 2022), despite burgeoning evidence supporting the transdiagnostic involvement of RNT in diverse symptoms (Hernández-Posadas et al., 2024; Jandrić et al., 2023). Therefore, the current study sought to bridge this gap. In addition, we explored the role of event-related rumination in externalizing psychopathology dimensions, whereas previous studies have primarily focused on internalizing psychopathology. Considering that increases in psychiatric symptoms during the pandemic were not confined to PTSD or internalizing problems but extended to a broad range, including mania (Russo et al., 2022), it is meaningful to show that engaging in a certain type of event-related rumination conveyed differential implications for subsequent adaptation, ranging from internalizing to externalizing psychopathology. Moreover, we provide longitudinal evidence that could overcome the directionality issue in previous cross-sectional studies. By utilizing a prospective two-wave design, we demonstrated that preceding cognitive processes influenced differences in subsequent symptom severity. In summary, our longitudinal findings suggest the importance of event-related rumination as a transdiagnostic RNT factor underlying various dimensions of psychopathology ranging from internalizing to externalizing symptoms.

With regard to clinical implications, we agree with Squires et al. (2022) that individual cognitive processes such as rumination are practical targets for psychological intervention (Watkins et al., 2011). It is natural and even human to overthink about what has gone wrong during highly stressful periods, as rumination is generally initiated with the intent of reducing the discrepancy between the desired but unachieved goal and the current state (Martin and Tesser, 2006). However, a problem begins when instrumental quality is lost, and rumination deteriorates into unproductive emotion-focused coping, masquerading as problem-focused coping (Matthews and Wells, 2000).

In this context, an emphasis should be placed on guiding individuals to *differently* attending to their internal experiences during difficult times for prevention and intervention. For example, rumination-based cognitive behavioral therapy has been successful in treating depression and PTSD (Schumm et al., 2022). Additionally, our results suggest that rumination-focused interventions targeting IR attenuation may be effective for those suffering from other symptoms, including anxiety, somatization, and anger. Furthermore, it would be meaningful to test the possibility of extending rumination-focused interventions into a transdiagnostic treatment protocol for a wider range of psychiatric disorders (c. f. unified protocol for transdiagnostic treatment of emotional disorder, UP; Barlow et al., 2017).

However, this study has some limitations. First, only self-report measures were used, which may have been influenced by response bias. In particular, it remains unclear whether event-related rumination is related to physiological and neural correlates, comparable to those of depressive rumination or worry (Steinfurth et al., 2017), which may explain the underlying mechanism between event-related rumination and symptomatic manifestation. Second, although it was based on the traditional classification (Achenbach et al., 2016), the distinction between internalizing and externalizing psychopathology (e. g., somatization, mania) may not exactly fit into the newly evolving taxonomy (e. g., Hierarchical Taxonomy of Psychopathology, HiTOP; Michelini et al., 2021). Furthermore, the relationship with more externalizing symptom dimensions such as substance abuse or antisocial behavior should be explored in future research. Third, there were only two measurement time points, thereby limiting the possibility of investigating longitudinal relationships over an expanded time frame. Therefore, it is necessary to examine whether differences in ruminative thinking have long-term effects on mental health. Finally, drop-out rate was relatively high, although attrition analysis on baseline data did not suggest selective attrition, which may cause bias in results. Considering that retention rate tends to be much lower in an online-based longitudinal study (Bull et al., 2004), future research needs to implement certain measures to improve retention rate at follow-up (e. g., appearance, order, and length of the questionnaire, or combination of automated and personalized techniques; Murray et al., 2013).

## 5 Conclusion

Nevertheless, to the best of our knowledge, this is the first study to longitudinally explore the mediating role of event-related rumination from a transdiagnostic viewpoint. Both types of event-related rumination were positively associated with perceived stress. However, IR exacerbated a wide range of subsequent psychiatric symptoms, except mania, whereas DR ameliorated depression, suicide, and anger symptoms over time. These results highlight the role of common cognitive processes, regardless of the specific symptom dimensions, which can be utilized to devise a feasible and effective transdiagnostic psychosocial intervention within, and probably outside, the pandemic.

## Data Availability

The raw data supporting the conclusions of this article will be made available by the authors, without undue reservation.
